# Longitudinal investigation of the resistomes in Swedish pig farms

**DOI:** 10.1038/s44259-026-00251-2

**Published:** 2026-07-23

**Authors:** Valeriia Ladyhina, Susanna Sternberg-Lewerin, Axel Sannö, Erik Bongcam-Rudloff, Johan Dicksved, Elisabeth Rajala

**Affiliations:** 1 Department of Animal Biosciences, Swedish University of Agricultural Sciences, Uppsala, Sweden; 2 Uppsala Antibiotic Center, Uppsala University, Uppsala, Sweden; 3 Department of Clinical Sciences, Swedish University of Agricultural Sciences, Uppsala, Sweden; 4 Department of Applied Animal Science and Welfare, Swedish University of Agricultural Sciences, Uppsala, Sweden

**Keywords:** Computational biology and bioinformatics, Microbiology

## Abstract

We conducted a longitudinal profiling of environmental resistomes and microbiomes from ten Swedish pig farms in a low–antimicrobial usage context. Samples were collected from pig pen environments and analysed using shotgun metagenomic sequencing. Resistome and microbiome profiles showed stronger temporal than farm-specific variation, with several age-associated trends. Age-related trajectories diverged between microbiome and resistome, indicating that resistance dynamics are shaped by factors beyond microbial succession. The highest relative abundance of resistance determinants was observed for tetracyclines, followed by aminoglycosides, macrolide–lincosamide–streptogramin antibiotics, beta-lactams, and folic acid synthesis inhibitors—drug classes commonly used in Swedish pig production. Resistome patterns were partially associated with phenotypic resistance profiles from previous studies, while analysis of antimicrobial usage alone could not fully explain the observed resistome. Overall, these findings suggest that additional factors beyond antimicrobial usage contribute to the persistence and dissemination of antibiotic resistance genes in pig farm environments.

## Introduction

Antimicrobial resistance (AMR) poses a major threat to human and animal health and the global economy and is recognised as one of the major global health challenges of the 21st century^1,2^. The animal sector is one of the leading consumers of antimicrobials, accounting for approximately 73% of global antibiotic use, primarily for disease management and as growth promoters in livestock^3,4^. For example, in Denmark in 2020, 76 tonnes of antibiotics were used for disease treatments (usage of antibiotics as growth promoters has been banned in the European Union since 2006) in pig farms, which is nearly twice the amount used for human medical treatments in the country during the same period (45 tonnes)^5,6^. Usage of antibiotics in livestock production promotes the selection and accumulation of resistant bacteria including zoonotic bacteria. It raises concerns for animal health but also constitutes an important threat to human health as it can lead to untreatable infections in humans^3,7^. It has also been suggested that food-producing animals may serve as a reservoir for resistant bacteria, potentially representing a greater source of resistance genes than humans^3,8^. Furthermore, the rising global demand for animal protein is projected to contribute to an 8% increase in antimicrobial use (AMU) in livestock production between 2020 and 2030^3,9^.

Recent surveillance of antimicrobial use in livestock positions Sweden among the countries with the lowest levels of antibiotic consumption in Europe, with a reported usage of 6.0 mg/kg in 2023, compared to the EU average of 45.1 mg/kg^10^. Approximately 70% of all antimicrobial treatments in Swedish livestock are administered individually, underscoring a targeted approach to therapy with a minimal reliance on group oral medication^10,11^. However, products with high-dose zinc oxide (ZnO), used in pig feed to prevent post-weaning diarrhoea in piglets, were withdrawn in the EU in June 2022, which subsequently led to an increased reliance on oral group-administered antibiotics to manage post-weaning diarrhoea (A. Werinder & C. Greko, poster at the 10th Symposium on Antimicrobial Resistance in Animals and the Environment, 30 June 2025)^12^. Although antibiotic use in Swedish pig production is generally low, substantial variation exists between farms and across production stages. The highest frequency of treatments is observed during the suckling period and decreases with the age of the pigs. In the context of the escalating AMR crisis, characterising the composition and dynamics of farm-level resistomes is critical. While AMU is a major factor driving the selection and spread of AMR in bacterial pathogens, the dynamics of the on-farm resistome are shaped by additional factors such as management practices, biosecurity, and microbial ecology. Swedish pig production is generally characterised by batch-wise management systems, age-segregated production units, controlled indoor climates maintained through heating and mechanical ventilation, and designated heated resting areas for piglets. In addition, Swedish farms typically implement high biosecurity standards, including all-in-all-out production, cleaning and disinfection between batches of growing pigs, restricted animal movement, and strict hygiene routines for personnel and visitors. Housing environments are commonly based on solid concrete floors, often with the addition of sections with slatted floors. Additon of straw daily is mandatory, mainly to improve animal welfare and in some systems also to be utilised as bedding material. Differences in environment, housing, and management may influence microbial persistence, environmental reservoirs, and consequently the longitudinal dynamics of the farm resistome. Therefore, Swedish pig farms, with their strict and low antibiotic usage and high biosecurity standards, offer a uniquely suitable setting for analysis of these influences. This study investigates the development of farm resistomes over a full production cycle at ten Swedish farrow-to-finish pig farms using environmental sampling of pig pens floor combined with shotgun metagenomic sequencing. This work lays the groundwork for identifying other, non-AMU risk factors that contribute to the emergence and persistence of antimicrobial resistance in agricultural environments.

## Results

We conducted a longitudinal metagenomic investigation of resident microbial communities across ten Swedish farrow-to-finish pig farms with low AMU. Environmental floor-associated samples were collected to characterise the farm-level resistome, which is shaped not only by pig faecal material but may also be influenced by other environmental contributors such as dust, feed and antimicrobial residues, rodents, insects and farm personnel. Thus, the sampled floor material represents an integrated environmental matrix reflecting both microbial circulation and the accumulation of antimicrobial resistance genes within the farm environment. Our investigation focused on longitudinal comparison of resistome diversity within and between farms by following a single pig batch per farm throughout one production cycle, sampled at six visits with approximately four-week intervals from early post-farrowing to shortly before slaughter.

### Antimicrobial usage

Antibiotic usage data revealed overall low antimicrobial usage in the investigated pig batches, with two farms reporting no antibiotic treatments during the study period (Supplementary Table 1). The term pig-treatment days was used to represent number of pigs and number of treatment days for each batch on each farm. Beta-lactams were most frequently used, on 8 out of 10 farms, ranging between 3 and 617 pig-treatment days (PTD) over the entire production cycle but predominantly during the first two months of the piglets’ lives. Additional antibiotics used over the production cycle included drugs from the aminoglycoside (3 farms, 6–34 PTD), folic acid inhibitor (4 farms, 1–9 PTD), macrolide-lincosamide-streptogramin (MLS, 4 farms 1–52 PTD) and tetracycline classes (1 farm with 364 PTD in total). Only one studied farm had two incidents of group antibiotic treatment with administration via water of MLS drug class, while all the other farms reported only injection-based individual treatments.

### Pig environment microbiota during the production cycle

**Fig. 1:**
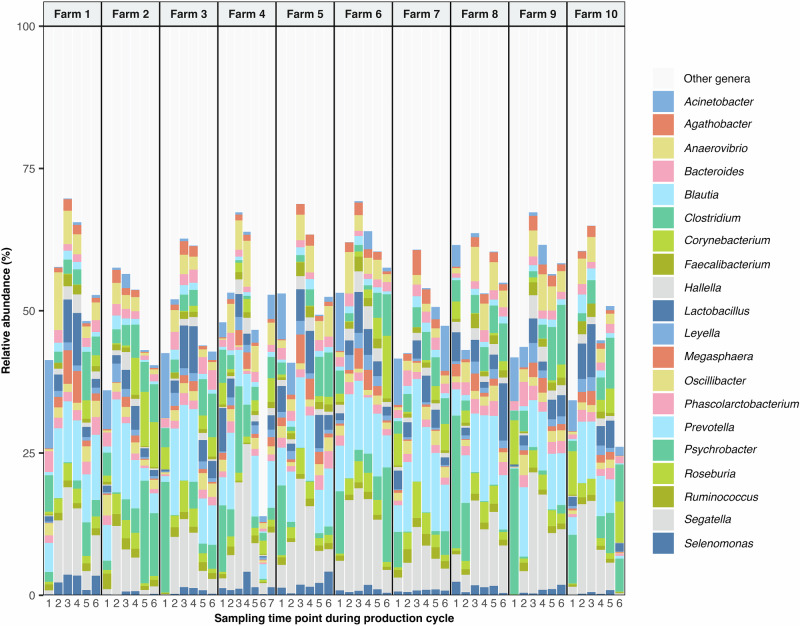
Temporal dynamics of the environmental microbiome from pig pens during one production cycle. Environmental microbiome data were derived from environmental samples collected from pig pen floors. Coloured genera represent the 20 most abundant genera detected across samples.

**Fig. 2:**
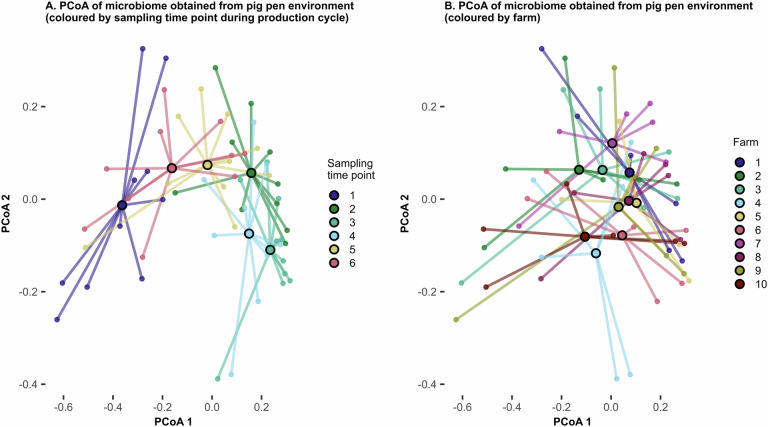
Principal coordinate analysis (PCoA) of pig pen environmental microbiome composition across 10 farms over production cycle (6 months). PCoA was performed using Bray–Curtis dissimilarities calculated from the 50 most abundant bacterial genera identified in the farm environment samples. **A** PCoA plot showing centroids representing different sampling time points (1–6), highlighting temporal patterns in microbial community structure. **B** PCoA plot with centroids representing individual farms (1–10), illustrating farm-level variation in environmental microbiota.

Analysis of the pig farm environmental microbiome showed that the number of detected bacterial genera remained relatively consistent across farms and sampling time points, ~4330 per sampling with 95% of found genera shared between all 10 farms at every sampling occasion, indicating a stable level of taxonomic richness in the studied microbiomes throughout the production cycle. Taking a closer look on taxonomy composition revealed differences between different sampling occasions and ages of pigs (Fig. 1). At the first sampling point, bacteria from the genera *Acinetobacter*, *Psychrobacter*, *Clostridium*, and *Bacteroides* were consistently ranked among the ten most abundant genera across all farms (Fig. 1). Bacteria belonging to genera *Prevotella* and *Segatella* were also found at the first sampling, however starting from the second sampling there was a visible increase in abundancies of these bacteria (Fig. 1). Principal coordinate analysis (PCoA) of the 50 most abundant bacterial genera revealed that pig age had a stronger influence on microbiome composition than farm of origin. Notably, the environmental microbiota at the first and sixth sampling points–corresponding to the period when piglets were still housed with sows and when pigs approached market age, respectively–showed greater similarity, suggesting age-related convergence in microbial profiles (Fig. 2).

### Resistome characterisation

Among all sampled farms, we identified 418 unique antimicrobial resistance genes (ARGs), that belong to 99 ARG gene families. Overall, 84% of the detected ARGs were identified with strict or perfect completeness when compared against reference databases. The detected ARGs are involved in resistance mechanisms to 16 classes of antibiotics, while only six antibiotic drug classes were used for treatments during the study period at the farms under investigation (Supplementary Table 1). Of these genes, 68.3% belonged to ARG gene families that were shared among all farms, most probably indicating the core resistome and reflecting a common selective pressure or microbial background. In contrast, only 4.5% of ARGs found in the sequenced data belong to ARG gene families discovered only at one farm.

**Fig. 3:**
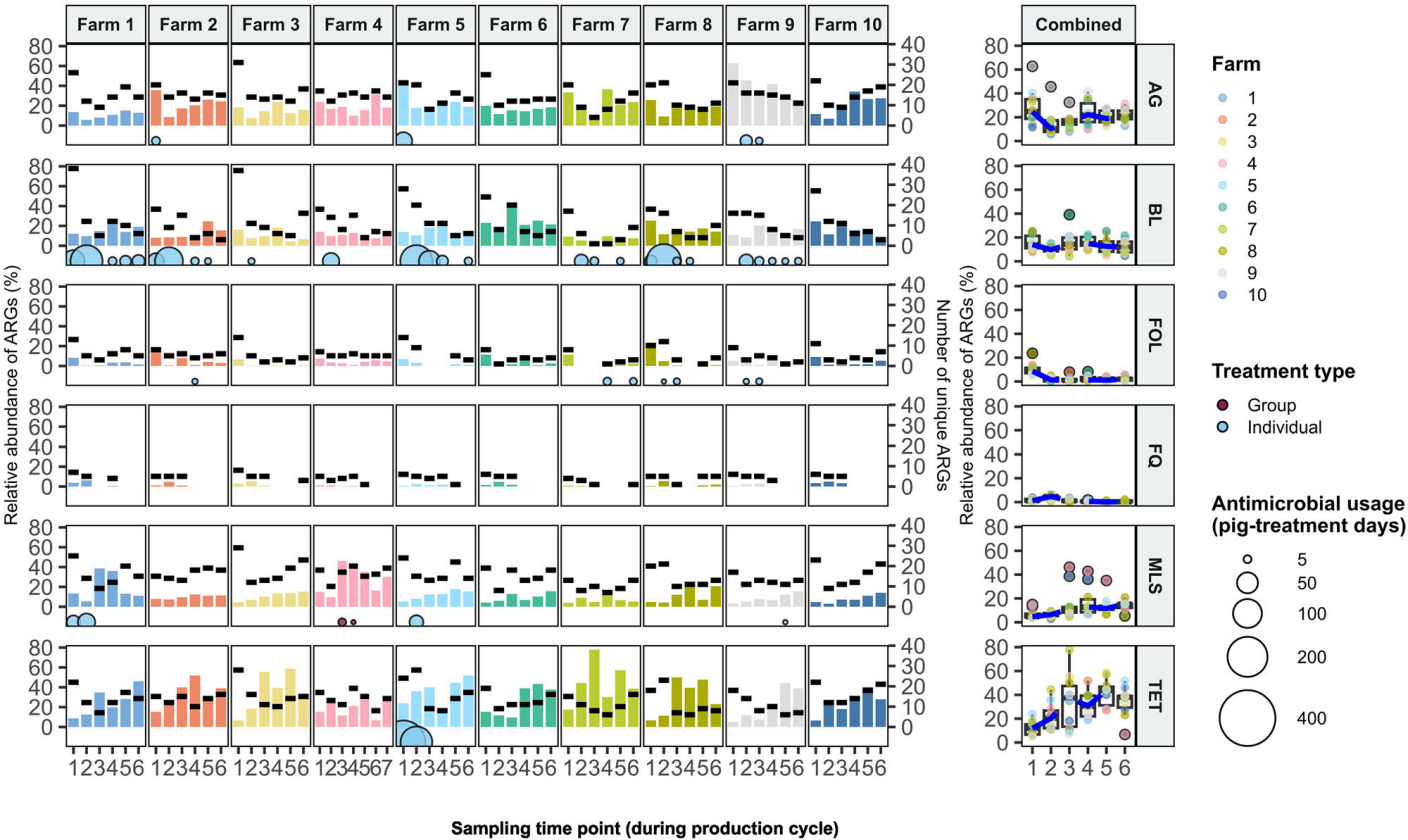
Longitudinal dynamics of antibiotic resistance genes detected in pig pens environment across pig farms over the production cycle (6 month), shown per farm and for all farms combined. Black horizontal segments indicate the number of unique antimicrobial resistance genes (ARGs) detected per sampling occasion, scaled to the secondary y-axis. Circles below the x-axis denote antimicrobial usage, with circle size proportional to the cumulative number of pig-treatment days prior to the sampling occasion and circle fill indicating treatment type (individual or group). Combined panels summarise resistome dynamics across all farms, with coloured points representing individual farms and dashed lines indicating median trends across farms. AG aminoglycosides, BL beta-lactams, FOL folic acid synthesis inhibitors, FQ fluoroquinolones, MLS macrolides-lincosamides-streptogramins, TET tetracyclines, AMU antimicrobial usage.

**Fig. 4:**
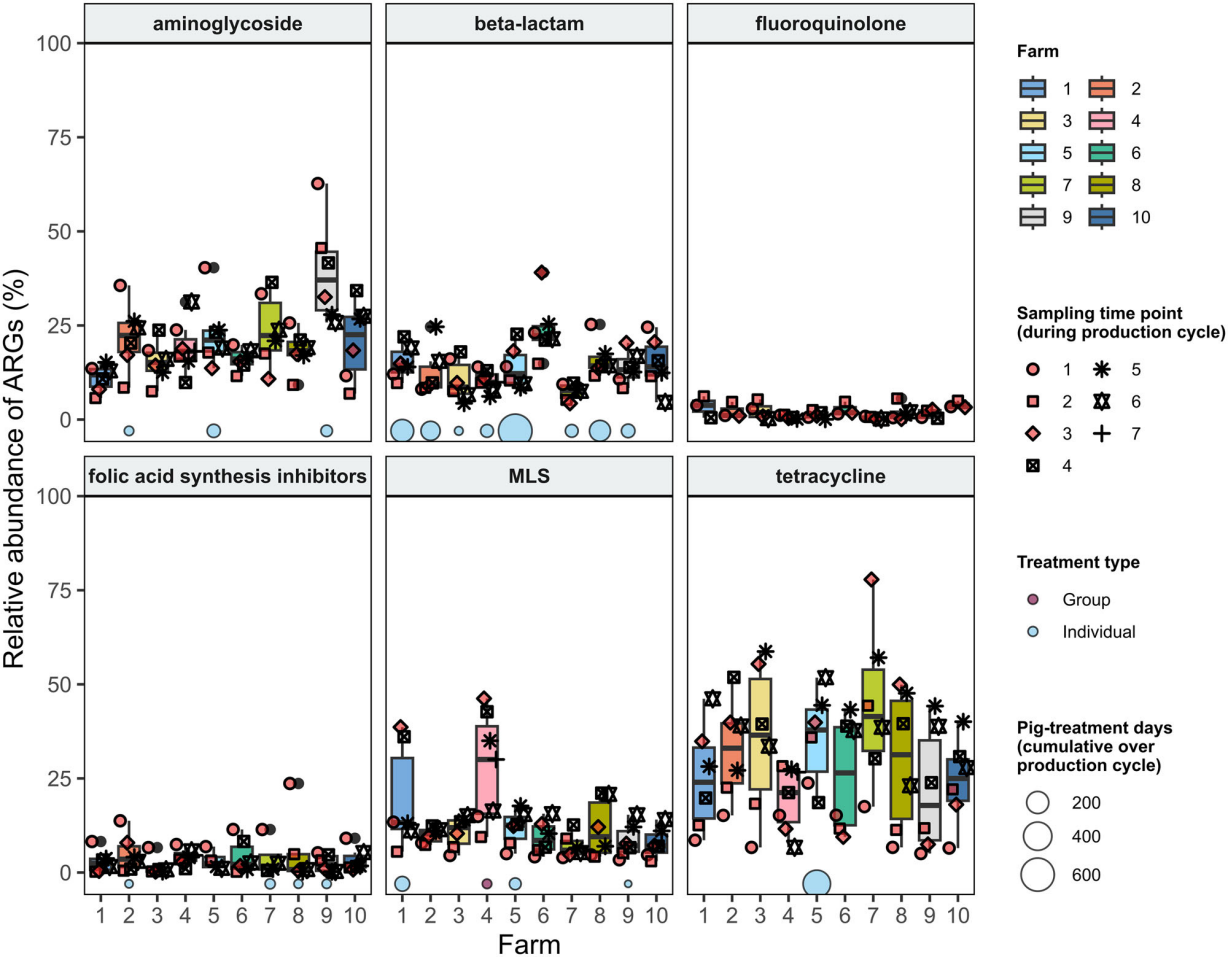
Farm-level distribution of pig pen environmental resistomes across the production cycle. Relative abundance of antimicrobial resistance gene (ARGs) detected in environments across 10 Swedish pig farms, grouped by the six most commonly used antimicrobial classes in pig production. Individual data points overlaid on the boxplots represent single sampling time points (sampling visits) during the production. Circles shown below the x-axis indicate cumulative antimicrobial usage per farm over the entire production cycle, expressed as pig-treatment days. AMU antimicrobial usage, MLS macrolide–lincosamide–streptogramin antibiotics.

**Fig. 5:**
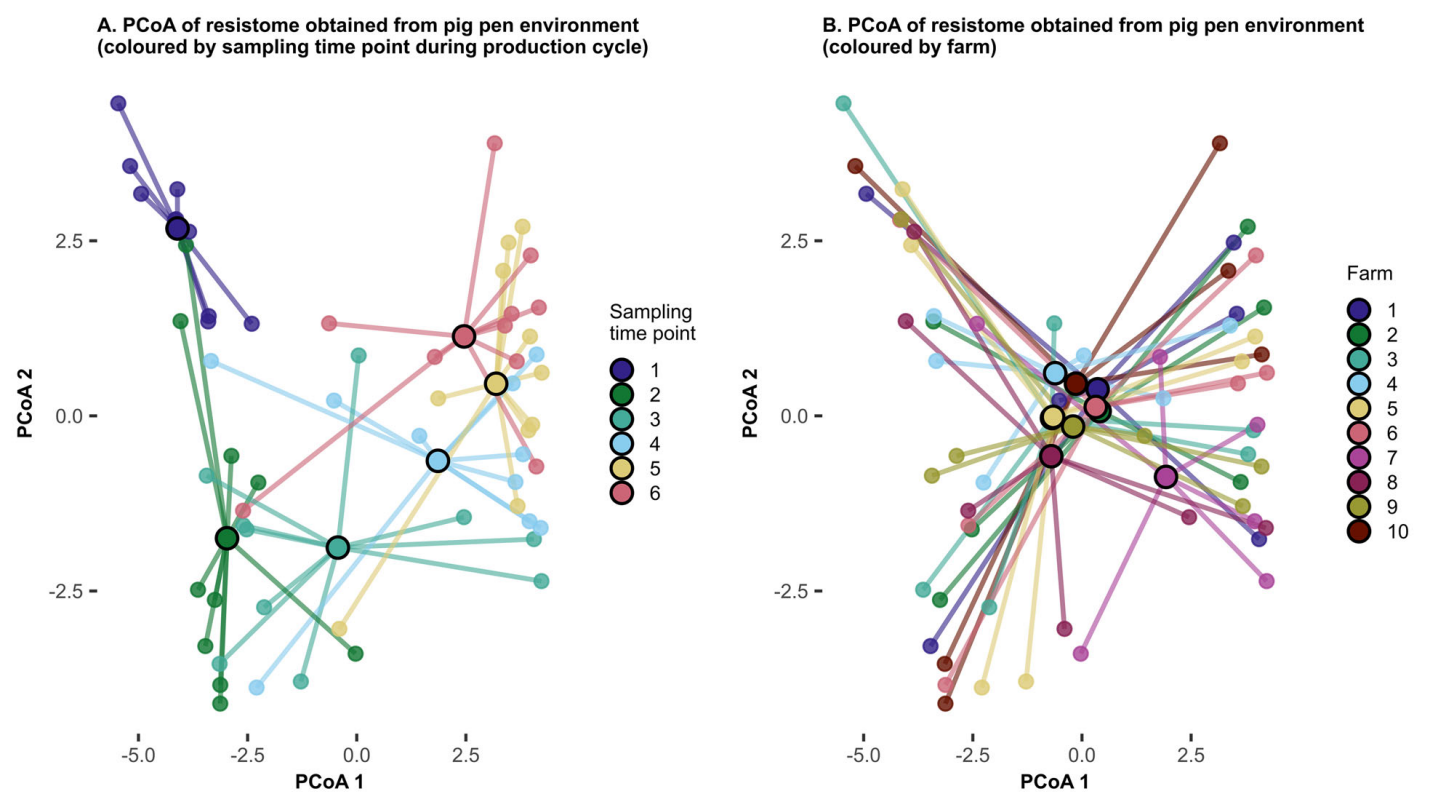
Principal coordinate analysis (PCoA) of pig pen environmental resistome composition across 10 farms. PCoA was performed using Jaccard dissimilarities. **A** PCoA plot showing centroids representing different sampling time points (1–6) during the production cycle, highlighting temporal patterns in resistome structure. **B** PCoA plot with centroids representing individual farms (1–10), illustrating farm-level variation in environmental resistome.

The comparison of farm resistomes was based on two measures: the variety of the resistome calculated as number of found unique ARGs per farm per sampling and the relative abundancies of detected ARGs calculated as the proportion of sequencing reads mapping to a specific ARG relative to the total number of ARGs-aligned reads within each sample (Fig. 3). The resulting resistome profiles revealed broadly consistent composition patterns over time across the ten pig farms studied. Across most farms, the diversity of unique ARGs peaked at the first sampling, followed by a marked decline by the second sampling and subsequent stabilisation, with some farms exhibiting a modest resurgence towards the end of the. production cycle (Fig. 3). For example, at all farms similar amounts of ARGs and fluctuation of relative abundances were found over the production cycle with resistance against the following antibiotic classes: aminoglycosides, beta-lactams, tetracyclines (Figs. 3, 4), broad-spectrum efflux pumps and glycopeptides (Supplementary Figs. 1, 2). A closer examination of longitudinal resistome dynamics revealed that the relative abundance of ARGs conferring resistance to MLS and tetracyclines increased progressively with the age of the pigs, particularly during the second half of the production cycle (Figs. 3, 4). Interestingly, some farms with MLS antimicrobial treatments exhibited higher MLS ARG abundances in the farm-level analysis (Fig. 4). However, the sample-level longitudinal analysis did not reveal a clear effect of individual treatment events on resistome dynamics, whereas group-administered MLS treatment appeared to be associated with an increase in the abundance of MLS resistance genes. Aminoglycoside resistance genes showed the highest abundance at the first sampling occasion, followed first by a decline and then an increase during the later stages of production (Fig. 3). For beta-lactam resistance genes, the diversity of unique ARGs decreased noticeably towards the later stages of the production cycle, while the overall relative abundance remained comparatively stable or showed only a minor reduction. Similarly, ARGs conveying resistance to folic acid synthesis inhibitors exhibited the highest relative abundance at the first sampling time point on nearly all farms, however further there was observed a progressive decline over the production cycle. The relative abundance of ARGs conferring resistance to fluoroquinolones were generally low and remained largely stable throughout the production cycle. In a subset of farms, these genes were not detected at later sampling time points. PCoA based on presence/absence profiles of ARGs demonstrated a clear effect of pig age, with samples from the first three sampling occasions clustering together. In contrast, farm-specific patterns were not evident, indicating limited influence of farm on overall resistome structure (Fig. 5).

## Discussion

In this study, we characterised the longitudinal resistome profile of the farm environment across ten Swedish pig farms over a full production cycle. Swedish pig production was selected as the focus due to its well-documented history of prudent antibiotic use and the predominance of individual, injection-based treatments^11^. Across the studied farms, antibiotic use was similarly low, with only two instances of group treatment (with MLS) in one farm reported. Given the low number of treatment events, the predominance of individual treatments but scarcity of detailed dosage data, dosage-based antimicrobial usage metrics were deemed less robust in this study setting. Under these conditions, pig-treatment-day-based measures provided the most consistent and comparable representation of antimicrobial exposure across farms and sampling time points. This aligns with national surveillance data^13^ and supports earlier findings that antimicrobial treatments in pigs are primarily administered during the early stages of the production cycle^11^. To our knowledge, this is the first study to capture the temporal dynamics of the environmental resistome in relation to a single pig cohort under low antibiotic pressure. This design provides a valuable opportunity to investigate baseline resistome development within high-biosecurity, low-antibiotic livestock systems.

Both pig gut and environmental microbiome serve as reservoirs of ARGs and it can influence both the abundance of ARGs as well as its composition^14,15^. Previous studies have suggested that the composition of the microbiome serves as a key determinant in shaping the resistome, influencing the emergence and persistence of AMR under selective pressure^16^. Several dynamic factors can affect the microbiome during the pig production cycle, including host age, diet, rearing environment, and antimicrobial exposure^17,18^. The microbial communities in this study included genera commonly associated with the porcine gut, such as *Prevotella*, *Segatella*, *Clostridium*, and *Lactobacillus*. In contrast, other detected genera such as *Acinetobacter* and *Psychrobacter* have been linked to environmental niches at pig farms: *Acinetobacter* was found to be predominant in the swine farm groundwater, while *Psychrobacter* was detected in higher abundance in straw-based housing systems^17,19–21^. The longitudinal analysis of the 50 most abundant bacterial genera revealed a strong shift in microbiome at the age of weaning. There was a visible increase in the abundance of *Prevotella* and *Segatella* genera from the second sampling, which was performed right after weaning in most of the farms. Both these genera belong to the *Prevotellaceae* family which has undergone several taxonomic changes over time, making detailed comparisons between older and more recent studies challenging^22^. Previous studies have reported similar findings, explaining the increase of relative abundancies of *Prevotella* by an abrupt separation from the sow that contributes to intestinal changes due to stress as well as a change of diet from milk to plant-based feed^20,23^.

PCoA revealed that samples collected during the first and final farm visits exhibited greater compositional similarity to each other than to those obtained at intermediate time points. This pattern contrasts with previous studies of the pig faecal microbiota, which have demonstrated that the post-weaning microbiome diverges markedly from that of unweaned piglets and tends to shift over time toward an adult-like profile resembling that of sows^24^. A likely explanation for this discrepancy lies in the nature of our sampling, which focused on the farm environment rather than direct faecal material. The environmental surfaces sampled at the initial visit were heavily contaminated by sow faeces—particularly in farrowing areas—thus driving the observed similarity to late-cycle samples, when older pigs contributed to environmental microbiota profiles. The reason for this strategy was that we wanted to explore the farm resistome rather than that of individual pigs and this study is the first step in that direction.

Resistome analysis revealed the presence of 401 ARGs conferring resistance to 16 classes of antibiotic drugs. The overall resistome profiles were relatively consistent across farms as well as in their temporal dynamics during the production cycle, both in terms of ARG composition and relative abundance. PCoA of the resistome revealed greater similarity among samples derived from pigs under three months of age, compared to those from older individuals. This pattern contrasts with age-related trends observed in the gut microbiome, where microbial composition typically undergoes a gradual and structured shift with maturation. The divergence between resistome and microbiome trajectories suggests that resistome composition in pigs may be shaped by factors other than microbial community structure alone. One possible explanation is the influence of AMU or other environmental risk factors, which may exert selective pressure independently of age-related microbial succession. Previous studies have shown that the impact of antimicrobial treatment on the resistome may be more pronounced than on the overall microbiome, indicating that ARG perturbations can occur independently of shifts in taxonomic composition^25^. The consistently higher diversity of unique ARGs observed at the first sampling, prior to any recorded antimicrobial exposure, mirrors findings by Gaire et al., who reported elevated ARG counts in 2-day-old piglets despite low microbiome diversity^26^.

In our study, the resistomes across all growth stages were dominated by ARG types conferring resistance to tetracyclines, aminoglycosides and MLS similar to previous longitudinal metagenomic studies^27,28^. These drug classes were also used for treatments on the study farms and reflect those commonly applied for therapeutic purposes in Swedish pig production, including the sporadic use of fluoroquinolones, as reported by national veterinary surveillance programs^13^. While farms in which pigs received individual MLS antibiotic treatments showed greater fluctuations in the relative abundance of MLS resistance genes, a closer examination of longitudinal ARG dynamics did not reveal a clear temporal association with individual treatment events. In contrast, the farm receiving group-administered MLS treatment displayed a pronounced shift in MLS-associated ARGs. This suggests that the limited number of individual antimicrobial treatments administered during this specific production cycle had little impact on short-term resistome dynamics under the studied conditions, whereas group treatment appeared to exert a more visible effect on the resistome. The results are in line with the assumption that non-treatment factors influence the resistance levels on the individual farm and that the effect of a single antibiotic treatment on resistance levels at the weaning stage may have a minor influence on the overall ARG abundance and prevalence^29^. Therefore, the temporal changes observed in the resistome are more likely attributable to other risk factors - such as environmental components, management practices or co-selection of genes–rather than direct antibiotic selection pressure.

Despite only one recorded tetracycline treatment across farms, high levels and an age-associated increase in tetracycline resistance were observed. This aligns with a meta-analysis showing that tetracycline resistance genes are among the most abundant in the porcine microbiome^30^. Baseline resistance can remain elevated for years even without direct exposure, likely due to co-selection from other antimicrobials/biocides or due to their persistence and environmental entrenchment^31,32^. In addition, tetracycline antibiotics are known to degrade slowly in manure and agricultural environments, where they can remain biologically active for extended periods, thereby exerting prolonged selective pressure even in the absence of recent treatments^33,34^. A similar picture was seen for ARGs against aminoglycosides and MLS, while there were almost no treatments with these antibiotics. Metagenomic surveys have frequently revealed a strong co-occurrence between tetracycline and macrolide resistance genes in pig faecal samples—for example, Munk et al.^16^ demonstrated that these ARG classes were consistently detected together across pig and broiler resistomes–providing ecological evidence for potential co-selection mechanisms. In our previous study on indicator bacteria from the same samples, resistance to tetracycline (9% of *E. coli*, 27% of *E. faecium*) and erythromycin (26% of *E. faecium*) were the most common resistance traits^35^. In contrast, beta-lactams were the most commonly used antibiotics on farms that reported antimicrobial use, yet the diversity of beta-lactam–associated ARGs decreased towards the later stages of the production cycle. Similar age-related declines were observed in our previous study^35^ as well as in previous analyses of resistance in *E. coli*^26,31^. This trend may be linked to the concentration of beta-lactam treatments during early life stages, followed by reduced selective pressure as animals mature.

While the reported treatments with folic acid inhibitors were mainly in the second half of the production cycle, we observed a decrease in the corresponding ARGs with aging of the pigs. Similar patterns were seen in our previous phenotypic study of these samples, with a decrease in resistance to sulfamethoxazole and trimethoprim in *E. coli* from the second sampling^35^. The low prevalence of ARGs conferring resistance to fluoroquinolones in our study is consistent with Swedish national surveillance data, as well as with the absence of any treatment records indicating the use of fluoroquinolones on the study farms^13^. The difference between the longitudinal patterns of the resistomes and the microbiomes in these low-AMU environments indicate that there are other risk factors that affect the development, spread and persistence of ARGs within these environments. This will be the focus of our future studies.

The resistome patterns differed from microbiome dynamics and could not be fully explained by antimicrobial use alone, suggesting that additional factors contribute to shaping the resistome. Moreover, temporal variation within farms exceeded differences observed between farms. Further investigation is needed to disentangle the relative contributions of antimicrobial usage, environmental factors, and management practices to resistome dynamics across the production cycle.

## Methods

### Sampling

The study had a longitudinal design in which one batch of pigs was followed throughout the entire production cycle on ten Swedish farrow-to-finish farms, as described in Ladyhina et al.^35^. Study farms were selected based on proximity to the Swedish University of Agricultural Sciences (≤1.5 h driving distance), use of a farrow-to-finish production system, and the feasibility of following a single pig batch for the full production cycle. Of the 13 farms meeting these criteria, 10 agreed to participate. One batch of pigs per farm was followed for approximately six months and sampled each ~4 weeks between February and September 2023; one farm (Farm 4) followed a slightly longer production cycle (~7 months). Sampling began when piglets were 1–2 weeks old and concluded 1–2 weeks prior to slaughter (~24 weeks old), except on Farm 10, where the final sampling occurred two days after pigs had been removed from the pens.

Samples were collected via sock/boot swabs as described previously^36^. In brief, floor samples were obtained by systematically walking through pig pens using sterile gauze socks, with sampling evenly distributed across all pens housing the studied batch of pigs to ensure representative spatial coverage. Up to four pairs of socks were collected at each farm and sampling occasion. During transport to the laboratory, samples were kept cooled at <4 °C.

### Sample processing

Immediately on arrival to the lab, the samples were diluted with 50 ml of sterile buffered peptone water and extracted with stomacher and subsequently concentrated via centrifugation. Finally, the pellet was resuspended in 15 ml of supernatant. All samples from each farm visit were pooled into one, resulting in a total of 61 samples (with one pooled sample per herd and sampling occasion) and stored at −80 °C.

### Ethics approval and consent

This work did not involve the use of animals or human participants. No ethical approval was required because no invasive animal procedures or experimental interventions were performed. Samples were collected non-invasively from the farm environment during routine production conditions without handling or disturbing the animals.

### DNA extraction

DNA was extracted according to the optimised method described in Ladyhina et al.^36^. In short, OMEGA Biotek kit (Omega Bio-tek, Norcross, GA, USA) was used according to the manufacturer’s instructions with minor adjustments: after bead beating the entire supernatant was diluted in double amount of RBB buffer and centrifuged in a MicroElute column.

### Sequencing

Library preparation was based on PCR-free NEBNext® Ultra™ IIDNA Library Prep Kit (Cat No. E7645) protocol and Illumina sequencing was performed by NOVOGEN UK Company Ltd. Samples were sequenced using 150 bp PE on NovaSeq X plus system (Illumina Inc., San Diego, CA, USA) with resulting depth of sequencing at least 80 M of PE reads per sample.

### Bioinformatic analysis

Scripts for the bioinformatic pipeline, based on our previous study^36^, are available at https://github.com/ValeriiaLadyhina/pigfarm-environmental-resistome-analysis.git. The pipeline included quality control and trimming of raw reads using fastp v0.19.5^37^, followed by removal of host contamination using bowtie2 v2.5.2^38^ and Samtools v1.3.1^39^ against the pig reference genome (*Sus scrofa* 11.1, NCBI). Taxonomic annotation was conducted on quality-filtered reads using Kaiju v1.10.1^41^ Metagenome assembly was performed with MEGAHIT v1.2.9^40^ and evaluated using metaQUAST v5.2.0^42^. with the RefSeq_nr database (2023_06_17). ARGs were identified using ABRicate v1.0.1^43^ against five databases: Argannot v5^44^, CARD v3.2.9^45^, ResFinder v2.3.2^46^, MEGARes v3.0^47^, and NCBI AMRFinderPlus (2024-05-02)^48^, applying minimum coverage and identity thresholds of 80%. To ensure comparability across databases, a standardised ARG nomenclature dictionary was constructed and made available via the GitHub repository. Resistome relative abundances were quantified by mapping reads back to assembled contigs using Bowtie2, processing alignments with Samtools, and calculating abundances of ARGs with Bedtools v2.25.0^49^ multicov normalised as reads per kilobase per million mapped reads values. Relative abundances were subsequently calculated by scaling the total ARG load within each sample to 100% and summarising abundances by antibiotic class.

Further analyses and data visualisation were performed in Microsoft R Open v4.5.1 using standard packages (cowplot, dplyr, ggforce, ggplot2, grid, paletteer, tidyr, tidyverse, and vegan). Microbiome PCoA was based on Bray–Curtis dissimilarities calculated from the 50 most abundant bacterial genera, while resistome PCoA was computed on ARG presence/absence data using the Jaccard index.

### AMU data collection and analysis

Information on antimicrobial usage in the sampled groups was collected from detailed farmer records. Farmers maintained a dedicated logbook documenting all treatments and daily events for the studied pig batches. AMU was quantified for each farm and pig batch for both the period preceding sampling at each location and the pigs’ entire production cycle, expressed as the total number of pig-treatment days (number of pigs treated x number of treatment days).

## Supplementary information

Supplementary Material

## Data Availability

AMU data are attached as Supplementary Table 1. Raw DNA sequence data from the 61 metagenomic samples across the 10 herds have been deposited in the European Nucleotide Archive under accession number PRJEB96782. The underlying code and ARG dictionary for this study is available in GitHub repository pigfarm-environmental-resistome-analysis and can be accessed via this link https://github.com/ValeriiaLadyhina/pigfarm-environmental-resistome-analysis.git.
