# Supplementary material for: Longitudinal investigation of the resistomes in Swedish pig farms
Source: NPJ Antimicrob Resist. 2026 Jul 23;4:45. doi: 10.1038/s44259-026-00251-2 (PMC13452822; doi:10.1038/s44259-026-00251-2)
Supplement: Supplementary file 1 [file 44259_2026_251_MOESM1_ESM.pdf]

Supplementary Table 1: Recorded antimicrobial use at ten Swedish pig farms during production cycle: number of days when there was treatment. * At this sampling polypeptidic antibiotic (colivet) was used for 8 days. Farms 6 and 10 didn't report any usage of antibiotics. [1]

Farm	Sampling	Drug	Pig-treatment days	AG	BL	FOL	MLS	TET	Type of Treatment
1	1	Penovet<br>Noropen<br>Tylan	47<br>11<br>19	0	58	0	19	0	Individual
1	2	Noropen<br>Bimoxyl<br>Penovet<br>Tylan	27<br>27<br>71<br>33	0	125	0	33	0	Individual
1	3	No Treatments Were Reported							
1	4	Penovet	6	0	6	0	0	0	Individual
1	5	Penovet	12	0	12	0	0	0	Individual
1	6	Penovet	16	0	16	0	0	0	Individual
2	1	Parofor<br>Penovet	6<br>28	6	28	0	0	0	Individual
2	2	Penovet	94	0	94	0	0	0	Individual
2	3	No Treatments Were Reported							
2	4	Penovet<br>Dofatrim	5<br>2	0	5	2	0	0	Individual
2	5	Penovet	3	0	3	0	0	0	Individual
2	6	No Treatments Were Reported							
3	1	No Treatments Were Reported							
3	2	Bimoxyl		0	3	0	0	0	Individual
3	3	No Treatments Were Reported							
3	4	No Treatments Were Reported							
3	5	No Treatments Were Reported							
3	6	No Treatments Were Reported							
4	1	No Treatments Were Reported							
4	2	Penovet		0	30	0	0	0	Individual
4	3	Tylan		0	0	0	5	0	Group
4	4	Tylan		0	0	0	1	0	Group

[1] AG, aminoglycosides: Hipracin, Parofor, Streptocillin; BL, beta-lactams: Ethacilin, Bimoxyl, Noropen, Penovet; FOL, folic acid synthesis inhibitors: Hippotrim, Dofatrim; MLS, macrolides-lincosamides-streptogramins: Tylan, Tylosin; TET, tetracyclines: Engemycin

4	5	No Treatments Were Reported							
4	6	No Treatments Were Reported							
4	7	No Treatments Were Reported							
5	1	Engemycin<br>Ethacilin<br>Parofor	241<br>421<br>32	32	421	0	0	241	Individual
5	2	Ethacilin<br>Engemycin<br>Tylosin	127<br>123<br>20	0	127	0	20	123	Individual
5	3	Ethacilin		0	51	0	0	0	Individual
5	4	Ethacilin		0	13	0	0	0	Individual
5	5	0							
5	6	Ethacilin		0	5	0	0	0	Individual
7	1	No Treatments Were Reported							
7	2	Penovet	19	0	19	0	0	0	Individual
7	3	Penovet	6	0	6	0	0	0	Individual
7	4	Hippotrim	5	0	0	5	0	0	Individual
7	5	Penovet	4	0	4	0	0	0	Individual
7	6	Hippotrim	4	0	0	4	0	0	Individual
8	1	Ethacilin	15	0	15	0	0	0	Individual
8	2	Ethacilin<br>Dofatrim	146<br>1	0	146	1	0	0	Individual
8	3	Ethacilin<br>Dofatrim	5<br>2	0	5	3	0	0	Individual
8	4	Ethacilin		0	4	0	0	0	Individual
8	5	No Treatments Were Reported							
8	6	No Treatments Were Reported							
9	1	No Treatments Were Reported							
9	2	Streptocillin<br>Dofatrim<br>Penovet	14<br>2<br>6	14	20	2	0	0	Individual
9	3	Streptocillin<br>Penovet<br>Dofatrim	4<br>3<br>3	4	7	3	0	0	Individual
9	4	Penovet	4	0	4	0	0	0	Individual
9	5	Penovet<br>Tylan	3<br>1	0	3	0	1	0	Individual
9	6	Penovet	4	0	4	0	0	0	Individual

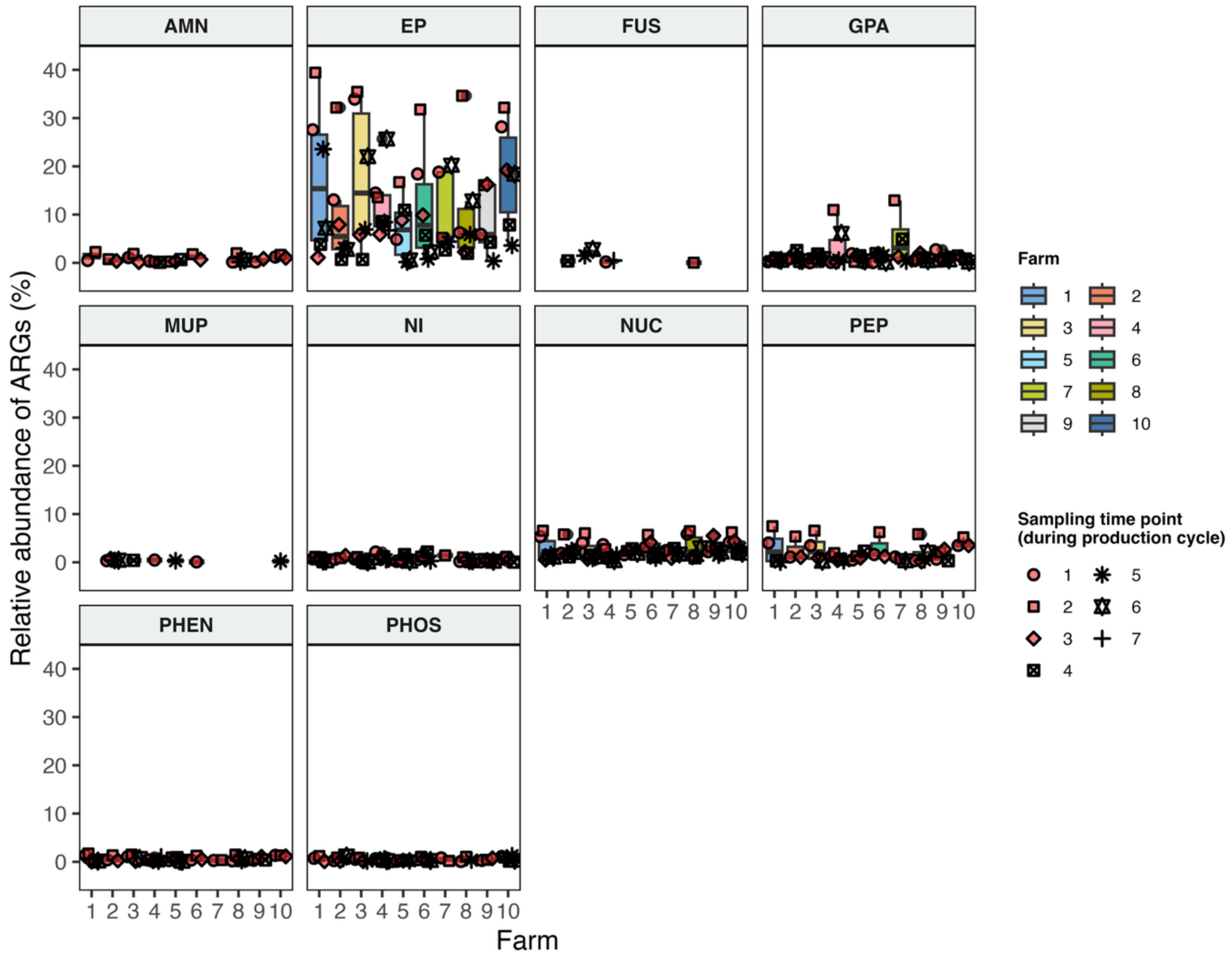

Supplementary Figure 1: Farm-level distribution of pig pen environmental resistomes across the production cycle.

Relative abundance of antibiotic resistance genes (ARGs) detected in environmental samples from 10 Swedish pig farms, grouped by additional antimicrobial classes represented in the pig pen environmental resistome. Individual data points overlaid on the boxplots represent single sampling time points (farm visits) during the production cycle. AMN, aminocoumarins; EP, broad-spectrum efflux pumps; FUS, fusidic acid; GPA, glycopeptides; MUP, mupirocin; NI, nitroimidazoles; NUC, nucleosides; PEP, peptide antibiotics; PHEN, phenicols; PHOS, phosphonic acid antibiotics.

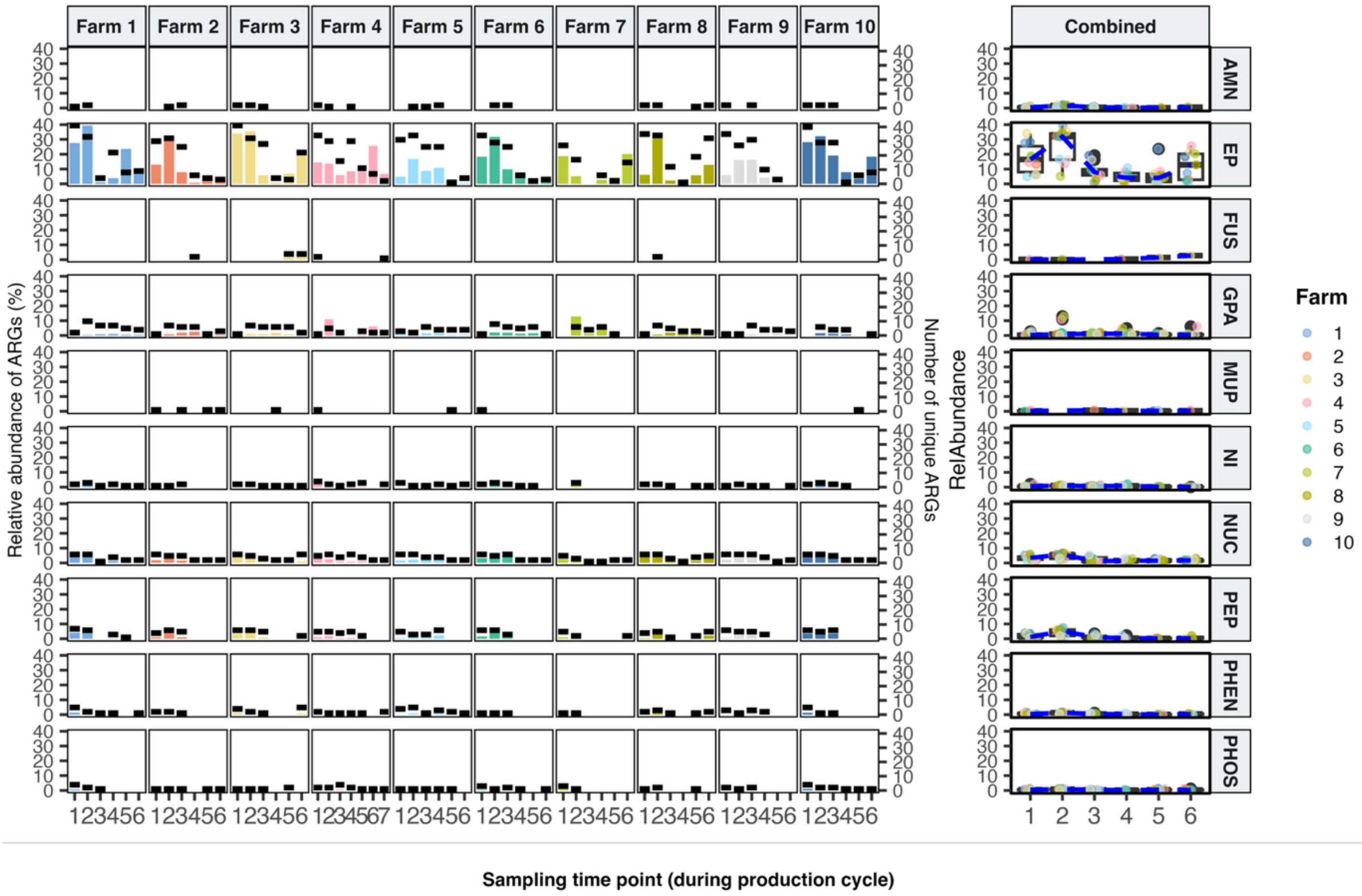

Supplementary Figure 2: Longitudinal dynamics of ARGs detected in pig pens environment across pig farms over the production cycle (6 month), shown per farm and for all farms combined.

Black horizontal segments indicate the number of unique ARGs detected per sampling occasion, scaled to the secondary y-axis. Combined panels summarize resistome dynamics across all farms, with colored points representing individual farms and dashed lines indicating median trends across farms. AMN, aminocoumarins; EP, broad-spectrum efflux pumps; FUS, fusidic acid; GPA, glycopeptides; MUP, mupirocin; NI, nitroimidazoles; NUC, nucleosides; PEP, peptide antibiotics; PHEN, phenicols; PHOS, phosphonic acid antibiotics.
